# Emergency Physician Attitudes, Preferences, and Risk Tolerance for Stroke as a Potential Cause of Dizziness Symptoms

**DOI:** 10.5811/westjem.2015.7.26158

**Published:** 2015-10-20

**Authors:** Mamata V. Kene, Dustin W. Ballard, David R. Vinson, Adina S. Rauchwerger, Hilary R. Iskin, Anthony S. Kim

**Affiliations:** * The Permanente Medical Group; † Kaiser Permanente Fremont Medical Center, Department of Emergency Medicine, Fremont, California; ‡ Kaiser Permanente San Rafael Medical Center, Department of Emergency Medicine, San Rafael, California; § Kaiser Permanente Northern California, Division of Research, Oakland, California; ¶ Kaiser Permanente Roseville Medical Center, Department of Emergency Medicine, Roseville, California; || University of California, San Francisco, Department of Neurology, San Francisco, California

## Abstract

**Introduction:**

We evaluated emergency physicians’ (EP) current perceptions, practice, and attitudes towards evaluating stroke as a cause of dizziness among emergency department patients.

**Methods:**

We administered a survey to all EPs in a large integrated healthcare delivery system. The survey included clinical vignettes, perceived utility of historical and exam elements, attitudes about the value of and requisite post-test probability of a clinical prediction rule for dizziness. We calculated descriptive statistics and post-test probabilities for such a clinical prediction rule.

**Results:**

The response rate was 68% (366/535). Respondents’ median practice tenure was eight years (37% female, 92% emergency medicine board certified). Symptom quality and typical vascular risk factors increased suspicion for stroke as a cause of dizziness. Most respondents reported obtaining head computed tomography (CT) (74%). Nearly all respondents used and felt confident using cranial nerve and limb strength testing. A substantial minority of EPs used the Epley maneuver (49%) and HINTS (head-thrust test, gaze-evoked nystagmus, and skew deviation) testing (30%); however, few EPs reported confidence in these tests’ bedside application (35% and 16%, respectively). Respondents favorably viewed applying a properly validated clinical prediction rule for assessment of immediate and 30-day stroke risk, but indicated it would have to reduce stroke risk to <0.5% to be clinically useful.

**Conclusion:**

EPs report relying on symptom quality, vascular risk factors, simple physical exam elements, and head CT to diagnose stroke as the cause of dizziness, but would find a validated clinical prediction rule for dizziness helpful. A clinical prediction rule would have to achieve a 0.5% post-test stroke probability for acceptability.

## INTRODUCTION

Dizziness is a common presenting symptom in the emergency department (ED) that is usually benign, but rarely the harbinger of stroke, particularly in the posterior circulation. Nationally, dizziness and vertigo symptoms accounted for 4% of ED visits overall in 2011.^1^ The total cost for these visits was estimated at $4 billion, which reflects the often-substantial resources involved in evaluating these patients in the ED with neuroimaging, specialty consultation, and hospital admission.^1^,^2^ Although dizziness-related ED visits and use of imaging studies during these visits increased from 1995–2004, there was no corresponding increase in the diagnosis of cerebrovascular disease among these patients.^3^ The prevalence of stroke was low in patients with dizziness as well: 3.2% of all ED patients with undifferentiated dizziness and only 0.7% of patients with isolated dizziness (dizziness, vertigo or imbalance without motor, sensory or language findings) were diagnosed with stroke or transient ischemic attack (TIA).^4^

Within this context, a clinical prediction rule to risk-stratify patients with dizziness could be useful in decision-making and resource utilization. Clinical prediction rules rely on readily obtainable historical, physical examination and clinical data to provide a standardized risk assessment for bedside decision-making. For example, the Pediatric Emergency Care Applied Research Network head injury clinical decision rule helps clinicians identify children at risk of clinically important brain injury after head trauma, in order to target the use of computed tomography (CT) imaging.^5^

As part of the process for developing a useful clinical prediction rule for dizziness in the ED, a better understanding of emergency physicians’ (EP) perceptions of their current practice and attitudes towards currently available diagnostic aids is crucial. Recently, a three-step bedside evaluation developed and tested by expert neuro-otologists (head-thrust test, gaze-evoked nystagmus, and skew deviation [HINTS]) has been proposed to clinically differentiate central from peripheral etiologies of vertigo, but its actual use in current emergency practice is unknown.^6^ Similarly, the required performance of a clinical prediction rule for dizziness evaluation to be clinically useful for EPs is also unknown. Therefore, we conducted a survey of EPs to assess their current practice, their attitudes and preferences for decision support, and to determine the specific risk thresholds that would make a clinical prediction rule useful in evaluating dizziness in the ED.

## METHODS

### Study Design and Population

We conducted a cross-sectional survey of EPs at the 21 EDs in the Kaiser Permanente Northern California (KPNC) system from August to October 2013. KPNC is an integrated healthcare delivery system that serves more than 3.7 million members; in 2013, there were nearly one million visits to the 21 community EDs systemwide.

We developed a comprehensive list of all EPs working across the system through individual contact with department leaders. EPs working more than five ED shifts per month were eligible to participate in this study. We excluded physicians who had been employed by KPNC for fewer than two months. These parameters were chosen to ensure the survey population included staff physicians with sufficient experience to understand the workings of the specific healthcare setting (resource availability, consultation services, etc). Study investigators were also excluded. These eligibility procedures were similar to prior EP survey studies conducted by our research group.^7^,^8^

The KPNC Institutional Review Board approved the study protocol and waived the requirement for written informed consent.

### Survey Content and Administration

We consulted the relevant literature on posterior circulation stroke and dizziness to develop the content of the survey**.** Specifically, we included items on specific history, exam findings, and clinical decision aids for evaluating stroke in patients with dizziness from the medical literature.^9^–^12^ We used answer choices with a 5-point Likert response format for agreement with statements (strongly agree, somewhat agree, neutral, somewhat disagree, and strongly disagree) and presented geometric series of probabilities for risk thresholds. Each question in the survey included “decline to answer” as a response option. The complete survey is available in an online appendix.

We pilot tested the instrument with the study project manager, the study investigators, the stroke neurologist (ASK) and four EPs not involved in survey construction, to ensure ease of use, relevance and comprehensibility. Responses from individuals who participated in pilot testing were not included in the analysis dataset. Based on the pilot testing, the initial questions were reorganized into sections that covered specific domains (e.g. the section eliciting the respondent’s suspicion for stroke based on specific exam and history findings), and we eliminated items from the section eliciting the specific targets for a candidate clinical prediction rule for EPs in order to focus on targets that were felt to be most relevant to practicing EPs in real time (e.g. admission and imaging decisions rather than estimating long-term stroke risk). We also modified the Likert response format choices to present uniform language across items from different sections of the survey instrument.

Email invitations for the electronic survey (SurveyMonkey, Palo Alto, CA) were sent to all 535 eligible EPs. We sent repeat invitations to non-respondents. Individuals who submitted partial or complete responses to the survey instrument received a $10 gift card; invitees who chose not to complete the survey could also write research staff to request a $10 gift card.

The survey contained clinical scenarios and questions designed to ascertain EP perceptions of their practice patterns and attitudes towards dizziness as a presentation of posterior stroke. Domains covered included self-reported use of bedside diagnostic tests, confidence in use of these tests, perceptions about utility of clinical decision aids to guide imaging, admission and disposition decisions, and demographic information about the respondents.

The first part of the survey was a clinical vignette. EPs were asked to estimate risk of stroke in two clinical vignettes of ED patients with dizziness: 1) a patient with undifferentiated dizziness with no other information provided; and 2) a patient aged 75 years with isolated dizziness, no neurologic findings on examination, and a normal electrocardiogram and hematocrit. Response choices were arranged in a geometric series with eight choices from 1/800 (0.125%) to 1 in 25 (4%), based on previously reported estimates placing the risk of stroke at 2–4% for undifferentiated dizzy patients and 0.5–1% of patients with isolated dizziness.^1^,^2^,^4^,^6^,^13^,^14^

In the second section, questions were designed to elicit the whether particular historical elements (15 symptom quality and vascular risk factors) and physical examination findings increased or decreased (greatly increase, somewhat increase, neutral, somewhat decreased, greatly decrease) EP’s suspicion for a stroke as a cause of dizziness, followed by questions about their perceived use of neuroimaging (CT, magnetic resonance imaging (MRI)) and specialist consultation for patients with dizziness (very frequently, frequently, occasionally, rarely, never). This section also queried EPs on perceived use and confidence in the use of common exam elements: HINTs, ABCD^2^, Epley maneuver and Dix-Hallpike testing (strongly agree, somewhat agree, neutral, somewhat disagree, and strongly disagree).^9^

In the third portion of the survey, respondents were queried about their areas of concern in the evaluation of patients with dizziness (overutilization of imaging, excluding stroke on clinical grounds alone) and their perceptions about the usefulness and appropriate target for a candidate clinical prediction rule (decision to obtain neuroimaging, disposition decision, or an assessment of the 30-day risk for disabling stroke).

To ascertain the requisite post-test probability in order for a dizziness-specific clinical prediction rule to be perceived as clinically useful to EPs, we asked respondents about necessary post-test probability of a clinical prediction rule targeting dizziness assuming a pre-test stroke probability of 3% (based on current evidence of stroke prevalence in patients with dizziness).^4^,^6^ We presented a geometric series of probability choices that included known estimated stroke risk for patients with dizziness, as well as acceptable post-test risk thresholds identified in studies of other conditions such as acute coronary syndrome and pulmonary embolism.^15^ Respondents were presented with choices in both probability and percentage formats (e.g., 1 in 100 and 1%).

Finally we collected demographic information such as age, gender and years in practice after residency.

### Data Analysis

ASK and MVK performed the statistical analysis using Stata (v13, College Park, TX). Descriptive statistics were tabulated. We excluded missing responses from the analysis. We evaluated the impact of longer tenure in practice post-residency on risk thresholds using the Wilcoxon rank-sum test on the acceptable risk threshold for a clinical prediction rule. Using these post-test probabilities and an estimate of the pre-test risk of stroke from the literature, we calculated the necessary likelihood ratio for a candidate clinical prediction rule to be considered clinically useful by EPs. Non-responders and responders were evaluated using the K-sample equality of medians test (tenure in practice) and the z-test (gender).

## RESULTS

### Characteristics of the Respondents

The response rate was 68% (366 respondents from 535 invitations). Respondents’ median time in practice after residency was eight years (range 1–40 years; interquartile range 4–14 years); 37% were female, and most were board certified in emergency medicine (92%) (Table 1). Non-responders (n=169) were 30% female, with median time in practice after residency of 10 years (range 1–38 years; interquartile range 5–16 years); board certification data is not available for non-responders. Bivariate analysis did not reveal significant differences between the responders and non-responders.

### Current Practices for Evaluating Dizziness in the ED

Respondents underestimated the stroke risk for patients with undifferentiated dizziness: 68% (n=247) estimated stroke risk at 0–1%, actual stroke risk 2–4%. For isolated dizziness, 50% of respondents overestimated the stroke risk at 2–4% (n=179) while 36% (n=131) correctly estimated stroke risk at 0.5–1%, actual stroke risk 0.5–1%. The actual stroke risk was drawn from previously reported estimates placing the risk of stroke at 2–4% for undifferentiated dizzy patients and 0.5–1% of patients with isolated dizziness.^1^,^2^,^4^,^6^,^13^,^14^

The impact of the specific description of dizziness, associated symptoms, and elements of the past medical history on the suspicion for stroke is illustrated in Table 2. Respondents reported that symptom quality influenced their suspicion for a central cause of dizziness, as did the presence of typical vascular risk factors such as age, diabetes, and hypertension.

**Figure 1:**
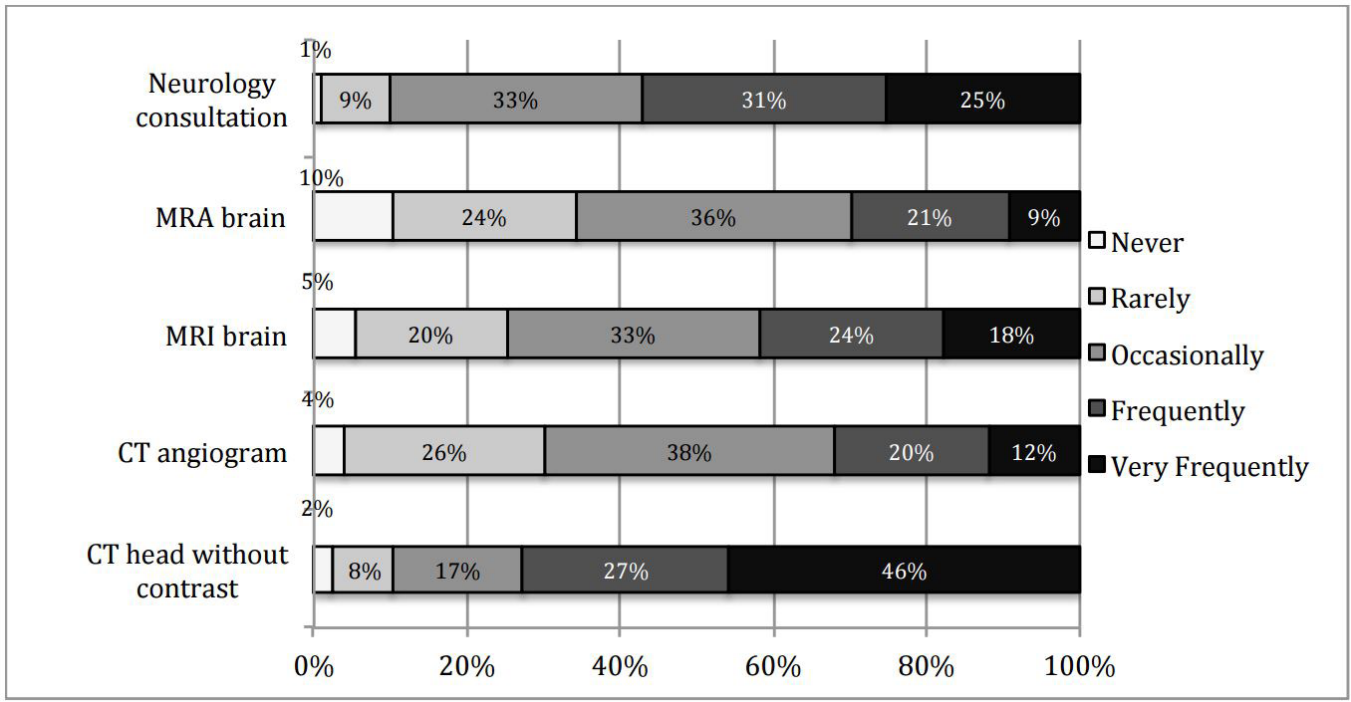
Current use of consultation and neuroimaging to evaluate dizziness in the emergency department^a^. ^a^Survey question 5: percentages indicate percent of respondents choosing a given answer. MRA-magnetic resonance angiogram MRI-magnetic resonance imaging CT-computed tomography

Current practice preferences for obtaining imaging and specialty consultation in dizziness patients are presented in Figure 1. Three-quarters of respondents reported frequent or very frequent use of head CT in the ED evaluation of dizziness (74%; n=260), although the same proportion of respondents agreed with the statement that CT was overused in the evaluation of dizziness (75% strongly or somewhat agreed; n=268).

**Figure 2:**
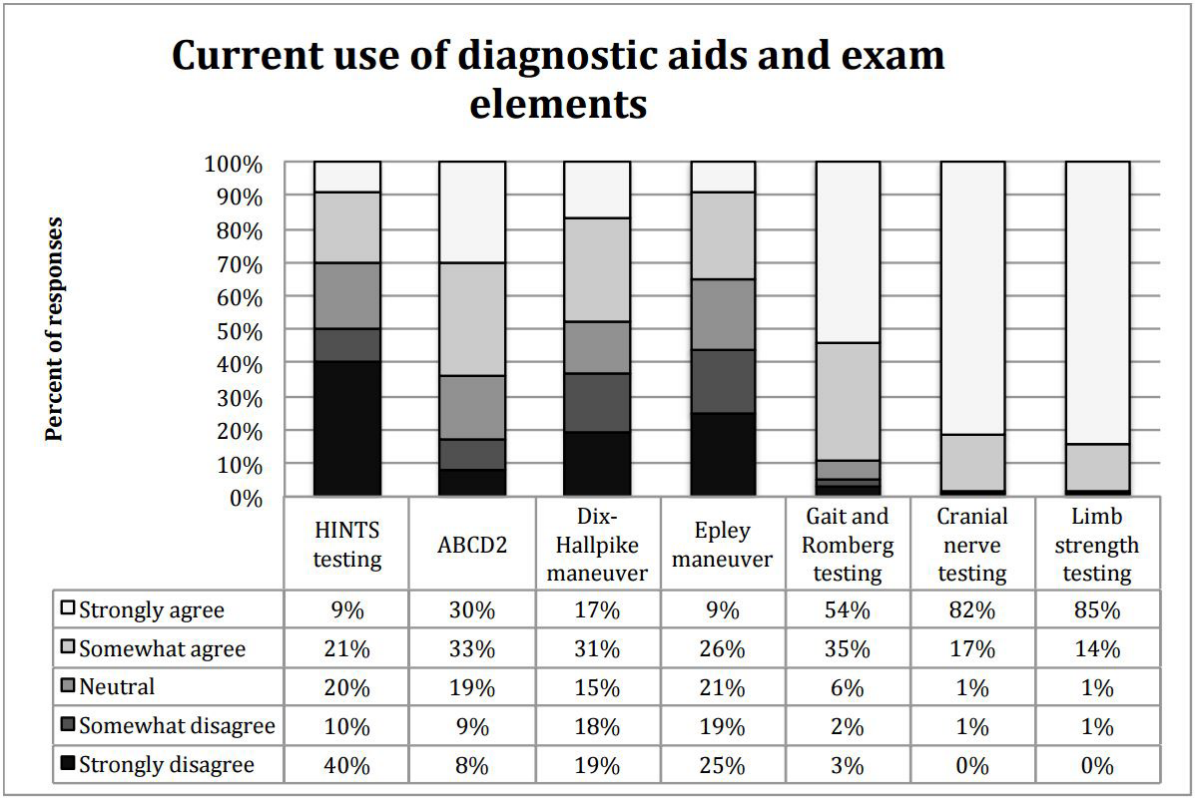
Respondents’ reporting of their perceived current use of bedside tests and clinical prediction rules to evaluate for posterior stroke among emergency department patients with dizziness^a^. ^a^Survey question 4, a-g, statement i HINTS-Head impulse, nystagmus, test of skew ABCD2-to predict 30 day risk of stroke after transient ischemic attach

**Figure 3:**
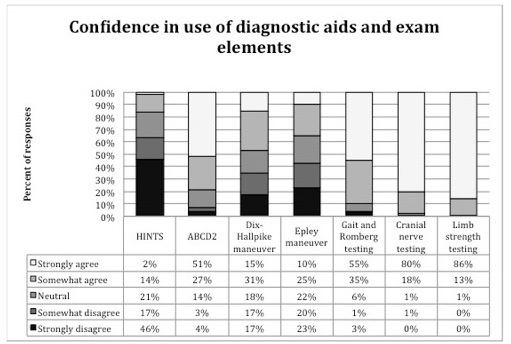
Agreement with feeling confidence in use of specific diagnostic aids and history and exam elements^a^. ^a^Survey question 4, a-g, statement ii HINTS-Head impulse, nystagmus, test of skew ABCD2-to predict 30 day risk of stroke after transient ischemic attack

Respondents’ agreement with statements about self-reported use of and confidence in using bedside diagnostic and physical examination findings and a commonly used clinical prediction rule for TIA (ABCD^2^) is shown in Figure 2. Confidence in applying these bedside diagnostic and physical exam tests and how often they were applied is reported in Figure 3. Respondents reported the lowest confidence in and likelihood of applying Dix-Hallpike and HINTS testing of the queried elements.

When EPs were asked whether they felt they were likely to use these specific tests if the tests were validated for the evaluation of stroke as a cause of dizziness, over 85% of respondents reported they were likely to use cranial nerve testing (89%), limb strength (86%) and gait evaluation (89%), while 58% reported they were likely to use Dix-Hallpike and 66% reported they were likely to use HINTS testing.

### Perceived Utility of Clinical Prediction Rules for Dizziness

EPs perceived estimating the immediate and 30-day risk of stroke, identifying candidates for hospital admission and identifying candidates for neuroimaging studies as appropriate targets for a clinical prediction rule (Table 3).

### Risk Thresholds for Stroke for a Clinical Prediction Rule for Dizziness

**Figure 4:**
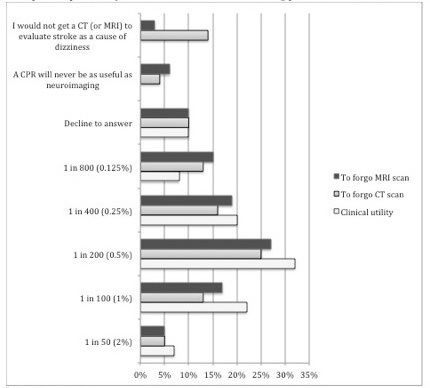
Ideal posttest probability for a CPR to be useful in evaluating patients with dizziness.^a,b^ ^a^9 missing responses ^b^Survey question 8: first two choices were not an option for the question about clinical utility MRA-magnetic resonance angiogram MRI-magnetic resonance imaging CT-computed tomography

Responses for target post-test probability for a clinical prediction rule on dizziness and stroke, including the non-numeric choices of “Decline to answer,” “I would not get a CT (or MRI) scan to evaluate posterior circulation stroke, ” and “A clinical prediction rule will never be as useful as neuroimaging” are presented in Figure 4, with the distribution of responses clustered around 0.25%–1% post-test probability of stroke, for those who identified a target post-test probability. Missing responses were low (0–3%), but we did observe a 10% decline-to-answer rate for questions relating to desired post-test probability, and this may have biased our results. Regarding using a clinical prediction rule to forgo neuroimaging in a patient with dizziness, 4% and 6%, respectively, of respondents marked that a clinical prediction rule would never be as useful as neuroimaging (CT or MRI). Fifty respondents (14%) reported that they would not obtain a CT to evaluate posterior circulation stroke as a cause of dizziness. Of those respondents who did indicate a numeric ideal post-test probability for a clinical prediction rule, at the median, respondents reported they would require a post-test probability of stroke of 0.5% for a clinical prediction rule to be clinically useful, to support not obtaining a head CT, or to support not obtaining MRI.

We further analyzed responses indicating a probability assuming linear distribution of the probabilities. First, we calculated mean probabilities: 0.65% (clinically useful), 0.58% (to support not obtaining a head CT), and 0.56% (to support not obtaining MRI). Using the stated 3% pre-test stroke probability, we generated likelihood ratios (LR) of 0.22, 0.19 and 0.19, respectively.

## DISCUSSION

The major findings of the study are: 1) in current practice, EPs self-report a greater reliance on symptom quality and basic elements of the neurologic examination than on more specialized bedside maneuvers such as Dix-Hallpike or HINTS testing to evaluate ED patients with dizziness; 2) stroke risk, hospital admission, and neuroimaging are all perceived as appropriate targets for a decision support with a validated clinical prediction rule; 3) the risk threshold preferred for clinical utility is on the order of 0.5% and is similar for these various decision targets.

Previous studies have reported that a patient’s description of dizziness symptoms is often inconsistent; hence, reliance on symptom quality to differentiate the cause of dizziness symptoms, as we saw in our study, may be misplaced.^12^ Tarnutzer et al cite multiple studies in which terms relating to the quality of dizziness (e.g. vertigo, lightheadedness or unsteadiness) were inconsistently applied by patients and provided little predictive value on stroke risk.^13^ Based on these data, we avoided focusing on the quality of dizziness symptoms (e.g. vertigo vs. lightheadedness), although we did note that respondents reported differing degrees of suspicion for stroke based on the description of dizziness. The presence of stroke risk factors and of motor, sensory, and speech findings increased EPs’ suspicion of stroke as a cause of dizziness, which are consistent with the typical diagnostic elements used in evaluating stroke more generally.

Of the bedside tests queried, respondents also indicated the lowest use of and confidence in applying HINTS. It is possible that the time required at the bedside to perform HINTS and Dix-Hallpike test or the frequency of use required to feel competent to apply these tests are a deterrent to their use in day-to-day clinical practice; however, this is unclear. Alternatively, respondents may not have been familiar with the interpretation or utility of these tests, especially HINTS, to evaluate for a central cause of dizziness. To date, no studies have assessed EP performance of HINTS testing; current literature reflects performance of the HINTS test by neurologists, neuro-ophthalmologists and neuro-otologists.^6^,^16^,^17^

Overall, EPs identified the decision to admit a patient, the decision to obtain neuroimaging, and the assessment of immediate and short-term stroke risk as useful targets for a clinical prediction rule. Our findings are consistent with previous research that EPs would find validated clinical prediction rules useful in clinical practice. 8,18,19 In a survey of priorities for clinical prediction rules, EPs ranked assistance in identifying serious or central cause of dizziness as a top priority.^18^ Despite this identified clinical need, no current clinical prediction rules have focused on the evaluation of dizziness. One proposed bedside aid to assess the risk of stroke, HINTS, has been reported to have an LR of 0.04 for excluding stroke, but was developed in a highly selected and high-risk subpopulation of dizziness patients (59.5% of this cohort had posterior stroke and all had been admitted to the hospital).^16^ Whether it performs as well in a lower-risk population or in the hands of front-line EPs remains uncertain.

The acceptable post-test probability of stroke (approximately 0.5%) among ED patients identified in this study is comparable to the risk thresholds for low-risk suspected acute coronary syndrome and for pulmonary embolism. That the post-test probability thresholds for these decisions were similar may reflect the baseline concern for identifying a posterior stroke or general risk tolerance for critical diagnoses. Pines and Szyld identified a 0.5% post-test probability of pulmonary embolism after D-dimer testing and a 1.1–1.5% post-test probability of acute coronary syndrome in low-risk patients after stress testing (SPECT and exercise echocardiogram), assuming a pre-test probability of 10%.^15^ However, whether this risk threshold could be reliably achieved via a dizziness clinical prediction rule based on readily available historical, physical exam and clinical data is uncertain.

We found that EPs tended to overestimate stroke risk associated with isolated dizziness, perhaps explaining the relatively frequent imaging use identified in previous studies.^1^ In one large healthcare system in 2008, 30% of patients evaluated for dizziness in the ED underwent either head CT or MRI.^20^ The more frequent use of CT (compared to MRI) may reflect the variable and limited availability of MRI as well as the initial priority of identifying non-ischemic causes of dizziness such as intracerebral hemorrhage; however, registry data suggests that only about 10% of all strokes are hemorrhagic in etiology, and in a review of patients with intracerebral hemorrhage, only 2.2% had dizziness as the primary symptom.^21^,^22^

A stroke and dizziness prediction rule could appropriately reduce resource utilization and radiation exposure for this common symptom. Given the low prevalence of stroke as a cause of dizziness (reported at 2–4% for all dizziness and 0.5–1% for isolated dizziness), a study to develop and validate a clinical prediction rule for dizziness and stroke may require identifying a higher-risk subpopulation to have a sufficiently high event rate in order to be feasible.^4^,^6^

## LIMITATIONS

This study had several potential limitations. Since the survey included EPs in a single integrated healthcare system in a distinct geographic region, our results may not generalize to other locations or practice settings. It is worth noting, however, that the KPNC system serves a heterogeneous population that is broadly representative of the surrounding population.^23^,^24^

We pilot tested the survey among a small group of physicians including the study investigators; this limited pretesting may have limited the opportunity improve the acceptability and reliability of the survey as applied to a larger group as well as the particular range of content domains that were covered. We chose the range of responses for risk thresholds for our instrument based on the literature on risk thresholds for other serious emergency conditions (acute myocardial infarction and pulmonary embolus), and we chose the specific symptoms and findings for our instrument based on previously reported factors that could influence the suspicion for stroke among patients with dizziness, but there may be other factors that were not included in our instrument that could influence an EP’s estimates for the risk of stroke in a given patient with dizziness.^15^ In constructing the survey we also chose to use the term dizziness rather than vertigo or lightheadedness because previous data has shown that specific descriptors are inconsistently used by patients and have limited prognostic value.^12^,^19^

The suboptimal response rate (68%), though similar to other surveys of EPs, subjects our results to non-responder bias.7,8,25 However, non-responders had similar demographic characteristics as responders (gender proportion and tenure in practice). We consulted the relevant literature on survey studies of EPs in developing the format and content to achieve validity but the survey relied on EPs’ self-reporting of their current practice as well as of their use of diagnostic tests and consultation, which might not reflect their true practice.^7^,^8^,^18^ Perceptions of how physicians think they practice may not reflect their actual practice and utilization patterns, but in a survey format, data on actual ordering and utilization could not be collected. Respondents did receive an incentive for thoughtful completion of the survey to mitigate survey fatigue and non-responder bias.

Although we pilot tested the instrument for ease of use and reliability, survey fatigue could have reduced the reliability of items that were elicited later in the survey. Similarly, anchoring bias in the clinical vignette questions and the questions about risk reduction may have influenced responses for subsequent questions. Probabilities were presented in two formats to mitigate differences in responses due to the method of presentations (e.g., 1 in 100 and 1%). Missing responses were low (0–3%). Ten percent of respondents marked “decline to answer” for the questions about desired clinical prediction rule post-test probabilities for clinical utility, forgoing CT and forgoing MRI; these responses and the responses “I would not obtain a CT (or MRI) to evaluate posterior circulation stroke” and “A clinical prediction rule will never be as useful as neuroimaging” were excluded from the numeric analysis of desired post-test probability.

Our use of a Likert response format follows more recent usage of 5-point answer choices, but the distance between the response choices cannot be assumed to be either continuous or equidistant, limiting the scope of possible statistical analyses. Carifio and Perla address the problems with conversion of Likert response format to a continuous variable for interpretation, especially for the interpretation of survey questions individually.^26^

Despite these limitations, we believe the survey provides insights into physician practice, preferences and attitudes for the evaluation of dizziness in the ED.

## CONCLUSION

EPs rely on history and physical exam elements over bedside diagnostic tests such as HINTS and Dix-Hallpike to evaluate ED patients with dizziness. Overall, respondents had a favorable view of the utility of a clinical prediction rule to assist in making decisions about neuroimaging and admission for patients with dizziness and possible stroke. A successful clinical prediction rule to assist in decision-making about neuroimaging or admission would require a reduction in the post-test probability of stroke to approximately 0.5% in order to be clinically useful to most respondents.

## Figures and Tables

**Table 1:** Demographic characteristics of 366 emergency physician (EP) respondents and 169 EP non-respondents at 21 emergency departments.

	Respondents	Non-respondents	

	n (%)	n (%)	
Gender^a^			
Female	133 (37)	51 (30)	p=0.11
Male	224 (63)	118 (70)	
Board certified in emergency medicine^a^			
Yes	332 (92)	Data unavailable	
No	25 (7)	Data unavailable	
Years in practice^a^			
Median	8 years	10 years	p=0.06
Interquartile range	4–14 years	5–16 years	

a 9 respondents did not answer this question.

**Table 2:** Impact of various historical and exam factors on the suspicion for stroke as cause of dizziness in the emergency department.

Finding^a^	Greatly increase	Somewhat increase	Neutral^b^	Somewhat decrease	Greatly decrease

	n (%)	n (%)	n (%)	n (%)	n (%)
Age over 45	59 (16)	256 (70)	40 (11)	7 (2)	1 (0.5)
Diabetes mellitus	111 (31)	230 (64)	19 (5.3)	0 (0)	1 (0.3)
Prior history of stroke	233 (64)	127 (35)	3 (0.8)	0 (0)	1 (0.3)
Suddenness of onset of dizziness symptoms	19 (5)	98 (27)	157 (43)	68 (19)	21 (6)
Spinning sensation	7 (1.9)	39 (11)	206 (57)	86 (24)	24 (6.7)
Constant dizziness, worsening with movement	24 (6.7)	140 (39)	113 (31)	73 (20)	13 (3.6)
Intermittent dizziness that resolves when not moving	5 (1.4)	22 (6.1)	64 (18)	166(46)	105 (29)
Associated nausea and vomiting	5 (1.4)	41 (11)	265 (73)	45 (12)	6 (1.7)
Hypertension at evaluation (blood pressure≥140/90 mmHg)	20 (5.5)	219 (60)	123 (34)	2 (0.6)	0 (0)
Nystagmus	15 (4.2)	68 (19)	197 (55)	66 (19)	10 (2.8)
Unilateral weakness	321 (88)	34 (9.4)	6 (1.7)	1 (0.3)	1 (0.3)
Unilateral sensory loss	297 (82)	51 (14)	9 (2.5)	5 (1.4)	2 (0.6)
Inability to walk	175 (48)	125 (34)	62 (17)	1 (0.3)	0 (0)
Double vision	282 (77)	68 (19)	13 (3.6)	0 (0)	1 (0.3)
Speech disturbance	328 (90)	26 (7)	7 (1.9)	1 (0.3)	1 (0.3)

a Among non-missing; range of missing responses: 2–10.

b Neutral=neither increase nor decrease.

**Table 3:** Potential targets for a clinical prediction rule for dizziness.

Target^a^	Strongly agree	Somewhat agree	Neutral	Somewhat disagree	Strongly disagree

	n (%)	n (%)	n (%)	n (%)	n (%)
To assess immediate stroke risk in ED patients with dizziness	245 (68)	98 (27)	13 (3.6)	3 (0.8)	3 (0.8)
To exclude stroke as a cause of dizziness in ED patients WITHOUT neuroimaging	276 (76)	69 (19)	12 (3.2)	3 (0.8)	1 (0.3)
To help decide whether to obtain neuroimaging in ED patients with dizziness	258 (71)	84 (23)	15 (4)	4 (1)	1 (0.3)
To help determine whether an ED patient with dizziness warranted admission	222 (61)	98 (27)	28 (7.7)	11 (3)	3 (0.8)
To assess the 30-day risk of disabling stroke in ED patients with dizziness	193 (53)	120 (33)	29 (8)	14 (1.9)	6 (1.7)

*ED*, emergency department

a Among non-missing; range of missing responses: 2–10.

## References

[1] Saber Tehrani AS, Coughlan D, Hsieh YH (2013). Rising annual costs of dizziness presentations to U.S. emergency departments. Acad Emerg Med.

[2] Newman-Toker DE, Hsieh YH, Camargo CA (2008). Spectrum of dizziness visits to US emergency departments: cross-sectional analysis from a nationally representative sample. Mayo Clinic Proc.

[3] Kerber KA, Meurer WJ, West BT (2008). Dizziness presentations in U.S. emergency departments, 1995–2004. Acad Emerg Med.

[4] Kerber KA, Brown DL, Lisabeth LD (2006). Stroke among patients with dizziness, vertigo, and imbalance in the emergency department: a population-based study. Stroke.

[5] Kuppermann N, Holmes JF, Dayan PS (2009). Identification of children at very low risk of clinically-important brain injuries after head trauma: a prospective cohort study. Lancet.

[6] Kattah JC, Talkad AV, Wang DZ (2009). HINTS to diagnose stroke in the acute vestibular syndrome: three-step bedside oculomotor examination more sensitive than early MRI diffusion-weighted imaging. Stroke.

[7] Ballard DW, Reed ME, Rauchwerger AS (2014). Emergency physician perspectives on central venous catheterization in the emergency department: a survey-based study. Acad Emerg Med.

[8] Ballard DW, Rauchwerger AS, Reed ME (2013). Emergency physicians’ knowledge and attitudes of clinical decision support in the electronic health record: a survey-based study. Acad Emerg Med.

[9] Navi BB, Kamel H, Shah MP (2012). Application of the ABCD2 score to identify cerebrovascular causes of dizziness in the emergency department. Stroke.

[10] Newman-Toker DE, Cannon LM, Stofferahn ME (2007). Imprecision in patient reports of dizziness symptom quality: a cross-sectional study conducted in an acute care setting. Mayo Clinic Proc.

[11] Stanton VA, Hsieh YH, Camargo CA (2007). Overreliance on symptom quality in diagnosing dizziness: results of a multicenter survey of emergency physicians. Mayo Clinic Proc.

[12] Tarnutzer AA, Berkowitz AL, Robinson KA (2011). Does my dizzy patient have a stroke? A systematic review of bedside diagnosis in acute vestibular syndrome. CMAJ.

[13] Navi BB, Kamel H, Shah MP (2012). Rate and predictors of serious neurologic causes of dizziness in the emergency department. Mayo Clinic Proc.

[14] Kim AS, Fullerton HJ, Johnston SC (2011). Risk of vascular events in emergency department patients discharged home with diagnosis of dizziness or vertigo. Ann Emerg Med.

[15] Pines JM, Szyld D (2007). Risk tolerance for the exclusion of potentially life-threatening diseases in the ED. AJEM.

[16] Newman-Toker DE, Kerber KA, Hsieh YH (2013). HINTS outperforms ABCD2 to screen for stroke in acute continuous vertigo and dizziness. Acad Emerg Med.

[17] Newman-Toker DE, Kattah JC, Alvernia JE (2008). Normal head impulse test differentiates acute cerebellar strokes from vestibular neuritis. Neurology.

[18] Eagles D, Stiell IG, Clement CM (2008). International survey of emergency physicians’ awareness and use of the Canadian Cervical-Spine Rule and the Canadian Computed Tomography Head Rule. Acad Emerg Med.

[19] Kerber KA, Fendrick AM (2010). The evidence base for the evaluation and management of dizziness. J Eval Clin Pract.

[20] Kim AS, Sidney S, Klingman JG (2012). Practice variation in neuroimaging to evaluate dizziness in the ED. Am J Emerg Med.

[21] Andersen KK, Olsen TS, Dehlendorff C (2009). Hemorrhagic and ischemic strokes compared: stroke severity, mortality, and risk factors. Stroke.

[22] Kerber KA, Burke JF, Brown DL (2012). Does intracerebral haemorrhage mimic benign dizziness presentations? A population based study. Emerg Med J.

[23] Gordon NP (2012). Similarity of the adult Kaiser Permanente membership in Northern California to the insured and general population in Northern California: Statistics from the 2009 California Health Interview Survey. www.dor.kaiser.org/external/chis_non_kp_2009/.

[24] Krieger N (1992). Overcoming the absence of socioeconomic data in medical records: validation and application of a census-based methodology. Am J Public Health.

[25] Backlund BH, Hopkins E, Kendall JL (2012). Ultrasound guidance for central venous access by emergency physicians in colorado. West J Emerg Med.

[26] Carifio J, Perla R (2007). Ten Common Misunderstandings, Misconceptions, Persistent Myths and Urban Legends about Likert Scales and Likert Response Formats and their Antidotes. J Soc Sci.

