# Supplementary material for: Emergency Physician Attitudes, Preferences, and Risk Tolerance for Stroke as a Potential Cause of Dizziness Symptoms
Source: West J Emerg Med. 2015 Oct 20;16(5):768–76. doi: 10.5811/westjem.2015.7.26158 (PMC4644052; doi:10.5811/westjem.2015.7.26158)
Supplement: Supplementary file 1 [file wjem-16-768-s001.pdf]

SUPPLEMENT: SURVEY CONTENT

CONSENT

Dear Emergency Physician,

We'd like to invite you to participate in a study of *** emergency physicians on the possible utility of clinical prediction rules (decision rules) for diagnosing posterior stroke presenting as dizziness in the Emergency Department. The goal of the study is to better understand how emergency physicians assess dizziness as a potential symptom of stroke.

This secure survey will take less than 10 minutes to complete. You may decline to answer any question or to participate in the study, as this study is completely voluntary. Nevertheless, we believe that your perspective and responses to all of the questions are important. All information obtained for this study will be kept confidential to the full extent permitted by law. Your name will not be revealed in any publication or report of results. Your decision whether or not to take part in the study will not affect your employment or compensation in any way.

Questions about your rights as a study participant, comments or complaints about the study may also be presented to the Institutional Review Board for the Protection of Human Subjects:

***

In appreciation for your valuable time and assistance, you will receive a $10 Starbucks gift card when we receive your completed questionnaire by mail or online. * The link to the survey attached is unique; please don't forward it to anyone else. It will be used only to confirm your completion of the survey so that we can send you a gift card in appreciation of your participation.

You will be eligible for this gift card whether you complete the entire survey or not.

---

*The approximately 500 people selected for this survey who are current Emergency Physicians are eligible for the gift card incentive, but some will choose not to participate. By law, you do not have to complete the survey to receive a gift card, but we hope you will do so. To request a gift card without doing the survey, print a copy this letter, write ***

PART I: Vignette

Q1

1) What is your estimate of the probability that a given emergency department (ED) patient with a chief complaint of dizziness is harboring a new posterior circulation stroke?

    a. <0.25%
    b. 0.25%
    c. 0.5%
    d. 1%
    e. 2%
    f. 4%
    g. 8%
    h. >8%

Q2

A 75-year-old male presents to the ED complaining of dizziness:

- He can't better define his symptoms than feeling dizzy, worse with moving
- **No unilateral weakness or sensory changes**
- **Mild nausea,** no vomiting or diarrhea
- **Wife last saw him normal yesterday before going to bed**
- **Hematocrit is normal** and no GI or GU bleeding symptoms
- **EKG is normal sinus rhythm at 80 bpm**
- **No associated syncope or seizure**

What is your estimate of the probability that posterior circulation a stroke is the cause for this particular patient's symptoms?

a. <0.25%
b. 0.25%
c. 0.5%
d. 1%
e. 2%
f. 4%
g. 8%
h. >8%

Section 2:

Q3: To what extent do the following findings affect your suspicion for stroke as a cause of dizziness in ED patients? Answer choices for each symptom or feature:

Greatly increase,
Somewhat increase
Neither increase nor decrease
Somewhat decrease
Greatly decrease

- **Historical factors**:

    a. Age over 45 years

    b. Prior history of stroke

c. Diabetes mellitus

- **Symptoms**:
    d. Suddenness of onset of dizziness symptoms
    e. Spinning sensation
    f. Constant dizziness, worsening with movement
    g. Intermittent dizziness that resolves when not moving
    h. Associated nausea and vomiting

- **Findings**:
    i. Hypertension at evaluation (blood pressure $\geq$ 140/90 mm Hg)
    j. Nystagmus
    k. Unilateral weakness
    l. Unilateral sensory loss
    m. Speech disturbance
    n. Double vision
    o. Inability to walk

Q4: Please rate your agreement with the following statements about clinical exam elements or prediction rule(s) to evaluate patients with dizziness.

Strongly agree,
Somewhat agree
Neutral
Somewhat disagree
Strongly disagree

a. HINTS testing (Head-Impulse—Gaze-evoked Nystagmus—Test-of-Skew)
    i. I use HINTS testing in evaluating patients with dizziness
    ii. I feel confident in my application of HINTS testing
    iii. I would use HINTS testing in evaluating patients with dizziness if it were well validated to identify stroke as a cause of dizziness

b. Dix Hallpike maneuver
    i. I use Dix-Hallpike maneuver in evaluating patients with dizziness
    ii. I feel confident in my application of Dix Hallpike maneuver
    iii. I would use Dix-Hallpike maneuver in evaluating patients with dizziness if it were well validated to identify stroke as a cause of dizziness

c. Epley maneuver for vertigo
    i. I use Epley maneuver in evaluating and treating patients with dizziness
    ii. I feel confident in my application of Epley maneuver
    iii. I would use Epley maneuver in evaluating patients with dizziness if it were well validated to identify stroke as a cause of dizziness

d. Gait and Romberg testing

i. I use gait and Romberg testing in evaluating patients with dizziness
ii. I feel confident in my application of gait and Romberg testing
iii. I would use gait and Romberg testing in evaluating patients with dizziness if it were well validated to identify stroke as a cause of dizziness

e. Cranial nerve testing
    i. I use cranial nerve testing in evaluating patients with dizziness
    ii. I feel confident in my application of cranial nerve testing
    iii. I would use cranial nerve testing in evaluating patients with dizziness if it were well validated to identify stroke as a cause of dizziness

f. Limb strength testing
    i. I use limb strength testing in evaluating patients with dizziness
    ii. I feel confident in my application of limb strength testing
    iii. I would use limb strength testing in evaluating patients with dizziness if it were well validated to identify stroke as a cause of dizziness

g. ABCD2 for TIA risk prediction
    i. I use ABCD2 in evaluating patients with dizziness
    ii. I feel confident in my application of the ABCD2 score
    iii. I would use ABCD2 in evaluating patients with dizziness if it were well validated to identify stroke as a cause of dizziness

Q5: How often do you use the following ancillary tests and services in the ED to evaluate patients with dizziness in whom you are concerned about posterior circulation stroke as a cause of their symptoms?

Very frequently
Frequently
Occasionally
Rarely
Never

a. CT head without contrast
b. CT angiogram of head and neck
c. MRI of the brain
d. MRA of head and neck
e. Neurology consultation

Q6: To what extent do you agree with the following statements? (Strongly agree, somewhat agree, Neutral, Somewhat disagree, Strongly disagree)

a. I am concerned about stroke as a cause of dizziness in ED patients.
b. I find it difficult to determine which patients may have stroke as a cause of dizziness in the emergency department

c. It is important not to miss any patients with stroke as a cause of dizziness in the emergency department

d. I am able to exclude stroke as a cause of dizziness in emergency patients with dizziness on clinical grounds alone

e. A dizzy patient with a normal exam can be safely discharged from the ED without a neuroimaging study.

f. Neuroimaging is more reliable than clinical findings in evaluating posterior circulation stroke as a cause of dizziness in the ED

g. CT scans are overused in the evaluation of dizziness in the ED

h. MRI scans are overused in the evaluation of dizziness in the ED

Section 4.

Q7: To what extent do you agree with the following statements?

Strongly agree,
Somewhat agree
Neutral
Somewhat disagree
Strongly disagree

a. I would find a clinical prediction rule helpful if it could help assess the immediate risk of stroke as a cause of dizziness in ED patients

b. I would find a clinical prediction rule helpful if it could help exclude stroke as a cause of dizziness in ED patients without neuroimaging

c. I would find a clinical prediction rule helpful if it could help decide whether to obtain imaging for an ED patient with dizziness

d. I would find a clinical prediction rule helpful if it could help determine if an ED patient with dizziness warranted hospital admission

e. I would find a clinical prediction rule helpful if it could help assess the 30-day risk of disabling stroke in ED patients with dizziness.

Q8. For the purposes of the next 3 questions, let us assume that the prior probability of a stroke among patients presenting to the ED with dizziness is 3%.

1. You apply a simple clinical prediction rule in the Emergency Department and obtain a post-test probability of stroke. How low would this post-test probability have to be for you to consider this prediction rule **clinically useful**?

   Pre-test probability of 3% → now post test probability of

   a. 2% (1 in 50)
   b. 1% (1 in 100)
   c. 0.5% (1 in 200)
   d. 0.25% (1 in 400)
   e. 0.125% (1 in 800)

2. You apply a simple clinical prediction rule in the Emergency Department and obtain a post-test probability of stroke. How low would this post-test probability have to be for you to consider **NOT obtaining a CT scan**?

   Pre-test probability of 3% now post test probability of
   a. 2% (1 in 50)
   b. 1% (1 in 100)
   c. 0.5% (1 in 200)
   d. 0.25% (1 in 400)
   e. 0.125% (1 in 800)
   f. A clinical prediction rule will never be as useful as neuroimaging.
   g. I wouldn't obtain a CT scan to evaluate posterior circulation stroke

3. You apply a simple clinical prediction rule in the Emergency Department and obtain a post-test probability of stroke. How low would this post-test probability have to be for you to consider **NOT obtaining an MRI scan**?

   Pre-test probability of 3% now post test probability of
   a. 2% (1 in 50)
   b. 1% (1 in 100)
   c. 0.5% (1 in 200)
   d. 0.25% (1 in 400)
   e. 0.125% (1 in 800)
   f. A clinical prediction rule will never be as useful as neuroimaging.
   g. I wouldn't obtain an MRI scan to evaluate posterior circulation stroke

Section 5.
Respondent characteristics

Q9 Gender: ☐1 Male ☐2 Female

Q10 Are you board certified in emergency medicine?

☐1 Yes ☐2 No ☐3 I don't know

Q11 How many years ago did you complete residency training?

____ Years

Q12 How many years have you worked at ***?

_____ Years

Q13 What is your primary practice location?

1. Emergency Department
2. Rapid Care or PIT (physician in treatment)
3. Ambulatory Clinic
4. Other, please specify ___________________________________

Q14

Please use the space below for any comments, or anything else you would like to tell us about your evaluation of patients with dizziness, or the usefulness of potential clinical decision aids in this area.
